# Genomic Characterization and Molecular Detection of a Novel *Carlavirus* Infecting *Angelica dahurica*: *Angelica carlavirus Virus*

**DOI:** 10.3390/microorganisms14061335

**Published:** 2026-06-14

**Authors:** Xiang Li, Yanhong Qin, Shuhao Lu, Shaojian Li, Suxia Gao, Guohao Xu, Xuemeng Li, Qi Liu, Zhaorong Chen, Fei Wang

**Affiliations:** 1 Henan Key Laboratory of Agricultural Pest Monitoring and Control, Key Laboratory of Integrated Crop Pests Management on Crops in Southern Region of North China, Ministry of Agriculture and Rural Affairs, Institute of Plant Protection, Henan Academy of Agricultural Sciences, Zhengzhou 450002, China; li15937316210@sina.com (X.L.); qinyanhong6040@163.com (Y.Q.); lushuhao0525@163.com (S.L.); lishaojianli@126.com (S.L.); gaosx78@126.com (S.G.); x1643011452@163.com (G.X.); limm022@163.com (X.L.); l15936995034@163.com (Q.L.); 2 College of Horticulture and Landscape Architecture, Tianjin Agricultural University, Tianjin 300392, China

**Keywords:** high-throughput sequencing, phylogenetic analysis, viral disease, genomic organization

## Abstract

*Angelica dahurica* (*A. dahurica*) is an important medicinal plant in China; however, its production is affected by viral infections, leading to reduced yields and quality. In this study, we identified a novel *carlavirus*, tentatively named *Angelica carlavirus virus* (AnCV), in the leaves of *A. dahurica* exhibiting mosaic and leaf crinkling symptoms. Notably, the complete genome of AnCV was 8562 nt long and contained six open reading frames, with a genomic organization typical of the genus *Carlavirus*. AnCV exhibited 44.5–57.8% nucleotide identity at the whole-genome level with known members of the genus *Carlavirus*. In the polymerase gene and coat protein regions, the highest nucleotide and amino acid identities were 59.4–60.0% and 46.5–55.8%, respectively, which were below the species demarcation criteria established by the International Committee on Taxonomy of Viruses for the genus *Carlavirus*. Importantly, 10 AnCV isolates clustered within subgroup I of the genus *Carlavirus*, forming a relatively distinct branch. Moreover, 119 of the 280 *A. dahurica* samples were positive for AnCV (detection rate of 42.58%). Our study revealed that AnCV is a novel member of the genus *Carlavirus* that infects *A. dahurica*, providing a theoretical basis for the monitoring and control of viral diseases in *A. dahurica*.

## 1. Introduction

*Angelica dahurica*, a perennial herb of the genus *Angelica* in the family Apiaceae, is widely used as a traditional Chinese medicinal plant. In particular, the roots of *A. dahurica* have a long history of application in China [1]. As one of the representative “genuine medicinal materials” of China, *A. dahurica* is mainly produced in Henan, Zhejiang, Sichuan, and Hebei. *A. dahurica* has several pharmacological effects, such as dispelling wind and dampness, relieving pain, opening the nasal passages, and reducing swelling and pus, and is widely used in the treatment of conditions such as colds, headaches, and toothaches. Moreover, *A. dahurica* is an important component of several classical prescriptions and Chinese patent medicines [2]. In addition to its traditional medicinal uses, *A. dahurica* extracts have economic value in the fragrance and cosmetics industries [3].

*A. dahurica* is commonly propagated by seeds, and this mode of reproduction facilitates the transmission of viruses to the next generation of plants [4]. Therefore, viruses can accumulate within the host over time, leading to reduced yield and quality [5]. To date, several viruses have been reported to infect *A. dahurica*, including the *tobacco ringspot virus* (TRSV) [6], *cucumber mosaic virus* (CMV) [7], and *angelica bushy stunt virus* (AnBSV) [8]. In field production, *A. dahurica* plants are often infected by one or multiple viruses, exhibiting symptoms such as yellowing, chlorosis, and leaf deformation, which impair photosynthesis and may result in plant death [9].

The genus *Carlavirus* belongs to the family *Betaflexiviridae* and was first reported in potatoes in 1923 [10]. Over the years, members of this genus have been identified in several plant families, often causing latent or mild infections, particularly in potatoes, ornamental plants, and horticultural crops such as lilies [11], chrysanthemums, and carnations [12]. According to the International Committee on Taxonomy of Viruses (ICTV), this genus currently comprises 61 recognized species. Representative members include the *carnation latent virus* (CLV) [13], *potato virus S* (PVS) [14], *potato virus M* (PVM) [15], *blueberry scorch virus* (BlScV) [16], and *cowpea mild mottle virus* (CPMMV) [17]. In addition, other members include *arracacha latent virus* (ALV), *artichoke latent virus M* (ArLVM), *butterbur mosaic virus* (ButMV) [18], *cardamine latent virus* (CaLV), *carrot virus S* (CarVS), *helleborus mosaic virus* (HeMV) [19], *hydrangea chlorotic mottle virus* (HCMoV) [20], *phlox virus B* (PhlVB), and *sedum latent virus* (SeLV). Notably, the genome of *Carlavirus* consists of a linear single-stranded positive-sense RNA (+ssRNA), typically approximately 8.0–9.0 kb in length, with a 5′ cap structure and a 3′ poly(A) tail. Generally, the genome contains six open reading frames (ORFs) that encode a replication-associated protein, triple gene block proteins (TGB1, TGB2, and TGB3), a coat protein (CP), and a cysteine-rich protein [21]. However, some recently reported members of the genus *Carlavirus* lack ORF6 and contain only five ORFs [22]. According to the classification criteria formulated by the ICTV for viruses of the genus *Carlavirus*, a potential species can be categorized as a different virus species if the nucleotide sequence similarity of the CP or polyprotein of the virus is <72% and the amino acid sequence similarity is <80% [23].

In this study, we performed genomic characterization of a novel *carlavirus* affecting the growth of *Angelica* in the field, tentatively named “*Angelica carlavirus virus* (AnCV)”. In addition, we analyzed the genomic structure, phylogenetic relationships, and molecular variations of the virus.

## 2. Materials and Methods

### 2.1. Test Sample

A total of 93 leaf samples were collected from cultivated *A. dahurica* plants exhibiting mosaic, chlorosis, and leaf crinkling symptoms in Henan Province, China, from June to July 2020. These samples were collected from four major production areas, including Yuzhou City (46/93), Wen County (35/93), Mengzhou City (7/93), and Dancheng County (5/93). For high-throughput sequencing, a leaf section of approximately 2 cm^2^ was excised from each of the 93 symptomatic *A. dahurica* leaf samples, and all leaf sections were pooled to generate one composite sample. The composite sample was used for total RNA extraction, library construction, and High-Throughput Sequencing (HTS) on an Illumina HiSeq platform by Berry Genomics (Beijing, China). The remaining tissue from each sample was stored at −80 °C for subsequent experiments.

Because the HTS analysis was intended for initial virus discovery in symptomatic plants, asymptomatic plants were not included in the sequencing pool. To further evaluate the occurrence of AnCV in field-grown *A. dahurica*, a total of 280 leaf samples were subsequently collected from independent cultivated plants across the surveyed production areas and tested by RT-PCR. Cultivar information was not consistently available for the collected samples and was therefore not used as a sampling criterion.

### 2.2. High-Throughput Sequencing

To screen for viruses in the collected material, small aliquots from individual leaves were mixed to generate one composite sample, which was sent to Berry Ge-nomics (Beijing, China) for high-throughput sequencing. Bioinformatics processing was conducted by Biowefind Co., Ltd. (Wuhan, China), and included read cleaning and sequence assembly. Adapter contamination and low-quality reads were removed with FASTP version 1.5.6c [24], after which clean reads were assembled into contigs using IDBA-UD version 1.1.1 [25]. The assembled contigs were then queried against the protein database through BLASTx on the NCBI website (https://www.ncbi.nlm.nih.gov/, accessed on 18 October 2020).

Sequencing output and quality-control statistics, including total raw reads, clean reads, clean bases, GC content, Q20, Q30, assembly statistics, sequencing depth, and virus-associated contig numbers, were summarized from the HTS report. Viral contigs were identified based on BLASTx and taxonomic annotation results, and contigs showing similarity to known plant viruses were retained for subsequent primer design and genome confirmation.

### 2.3. Genome Assembly and Sequence Analysis of AnCV

Primers were developed from the contigs obtained by high-throughput sequencing (Table 1) and synthesized by Sangon Biotech Co., Ltd. (Shanghai, China). Total RNA was isolated from positive samples with a Spin Column Plant Total RNA Purification Kit (Sangon Biotech, Shanghai, China). First-strand cDNA was prepared from RNA templates using a PrimeScript™ II First Strand cDNA Synthesis Kit (Takara, Dalian, China) and stored at −20 °C until use. Overlapping genomic regions of AnCV were amplified by reverse transcription polymerase chain reaction (RT-PCR). Each 20 µL PCR contained 10 µL of 2× Taq Master Mix, 0.5 µL of each primer (10 µM), 1 µL cDNA, and ddH_2_O. Cycling was performed with an initial denaturation at 95 °C for 5 min; 35 cycles of 95 °C for 30 s, 55 °C for 30 s, and 72 °C for 1–2 min; and a final extension at 72 °C for 10 min. Amplicons were held at 4 °C. The 5′ and 3′ terminal sequences were determined by rapid amplification of cDNA ends (RACE) with a SMARTer 5′ and 3′ RACE Kit (Takara Biotechnology Co., Ltd., Dalian, China). PCR products were purified, ligated into the pMD19-T vector (Takara Biotechnology Co., Ltd., Dalian, China), introduced into *Escherichia coli* TG1 cells, and sequenced Sequencing was performed by Sangon Biotech Co., Ltd. (Shanghai, China). Overlapping fragments were assembled and analyzed in DNAMAN 7.0. ORFs were predicted with NCBI ORF Finder (https://www.ncbi.nlm.nih.gov/orffinder/ accessed on 20 October 2025), and the complete genome sequence was deposited in GenBank.

### 2.4. Phylogenetic Analysis

Briefly, the complete nucleotide sequences of AnCV isolates and full-length genome sequences of members of 12 genera (*Tepovirus*, *Vitivirus*, *Wamavirus*, *Prunevirus*, *Citrivirus*, *Trichovirus*, *Chordovirus*, *Ravavirus*, *Foveavirus*, *Robigovirus*, *Sustrivirus*, and *Carlavirus*) were retrieved from NCBI. Phylogenetic trees were constructed using MEGA 11.0 for taxonomic identification. In addition, the replicase (polymerase genes) and CP genes of the virus were selected to construct separate phylogenetic trees, with foveavirus (MW323519.1) used as the outgroup. In particular, trees were generated using the maximum likelihood method with 1000 bootstrap replicates. Complete-genome and gene-level alignments and identity calculations were carried out in DNAMAN 7.0.

### 2.5. Recombination Analysis of AnCV Isolates

Recombination analysis of the complete genome sequences of the 10 AnCV isolates was performed using RDP version 4.0. Potential recombination events were evaluated using seven methods implemented in the software: RDP, GENECONV, BootScan, MaxChi, Chimaera, SiScan, and 3SEQ. According to the predefined criteria, a recombination event was considered significant when the associated *p*-value was <1.0 × 10^−5^ and the event was detected by at least five of the seven methods. Conversely, recombination events with *p*-values > 1.0 × 10^−5^ were regarded as putative or potential recombination events.

### 2.6. RT-PCR Detection of AnCV

RT-PCR was used to amplify the target viral sequences in the samples, followed by purification of the PCR products and Sanger sequencing (Sequencing was performed by Sangon Biotech Co., Ltd., Shanghai, China). Finally, the obtained sequences were aligned and analyzed using DNAMAN 7.0 to assess molecular variation among viral isolates.

## 3. Results

### 3.1. High-Throughput Sequencing Data Analysis

HTS of the RNA-seq library generated 65,382,886 total raw reads. After quality control, 65,382,886 clean reads were retained, corresponding to a clean-read ratio of 100.0% and 9,789,414,344 clean bases. The GC content of the clean data was 46.14%, and the Q20 and Q30 values were 99.25% and 96.35%, respectively. De novo assembly generated 36,472 contig sequences with a total length of 24,231,638 bp. The assembly GC content was 41.94%, the N content was 0%, and the assembly sequencing depth was 403×.

For taxonomic annotation, 35,276 contigs with a total length of 23,484,074 bp were analyzed, of which 22,527 contigs, accounting for 63.86% of the total contigs, were classified. The total length of the classified contigs was 17,453,312 bp, accounting for 74.32% of the annotated contig length. Among the classified sequences, 326 contigs, containing 383 predicted ORFs and totaling 221,181 bp, were assigned to viruses. Sixteen virus-associated contigs showed similarity to members of the genus *Carlavirus*. The lengths of these contigs ranged from 218 to 2112 bp, with the highest sequence identity of 64.71% to *Ligularia sibirica* carlavirus (GenBank accession No.: OP783969.1).

Because the HTS report did not provide a per-base read-mapping profile for the AnCV genome, AnCV-specific coverage uniformity was not directly calculated from the sequencing report. Therefore, the complete genome sequence of AnCV was further confirmed by overlapping RT-PCR and 5′/3′ RACE.

### 3.2. Genome Organization of AnCV

**Figure 1:**
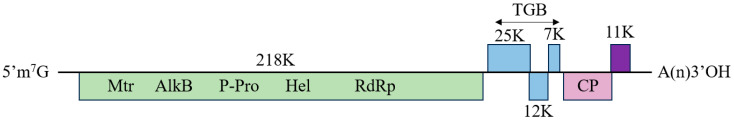
AnCV genome structure diagram.

A total of 10 sequences were obtained using RT-PCR and 5′/3′ RACE, with a complete genome length of 8562 nt (GenBank accession numbers: PZ059977 and PZ117032–PZ117040) and a poly(A) tail at the 3′ end. In addition, we predicted the ORFs in the genome using the ORF Finder tool available on the NCBI website (https://www.ncbi.nlm.nih.gov/orffinder/ accessed on 30 October 2025). Notably, the virus contained six ORFs (Figure 1), a structural feature consistent with the typical genome organization of members of the genus *Carlavirus*. ORF1 was located at positions 79–5988 nt and encoded a viral replicase protein consisting of 1969 amino acids, with a predicted molecular weight of approximately 218 kDa. Importantly, this replicase protein contains conserved domains, such as methyltransferase, RNA helicase, and RNA-dependent RNA polymerase (RdRp), which play key roles in viral RNA replication and transcription. In addition, the triple gene block (TGB) region consists of ORF2 (230 aa), ORF3 (108 aa), and ORF4 (64 aa), which encode proteins involved in viral movement between host plant cells. ORF5 (298 aa) encodes the CP, which is the major structural component of viral particles. ORF6 (109 aa) encodes a cysteine-rich nucleic acid-binding protein (NABP).

Further motif analysis revealed several conserved functional features in the AnCV-encoded proteins. The ORF1-encoded replicase contained a conserved RNA-dependent RNA polymerase catalytic GDD motif at amino acid positions 1842–1844, corresponding to nucleotide positions 5602–5610 of the AnCV genome. The surrounding conserved sequence, ICFAGDDMCASK, was located at amino acid positions 1838–1849, supporting the annotation of this region as the RdRp domain. The ORF2-encoded TGB1 protein contained a P-loop/NTP-binding-like motif, GAGKSS, at amino acid positions 31–36, corresponding to nucleotide positions 6110–6127. The ORF3- and ORF4-encoded TGB2 and TGB3 proteins contained hydrophobic regions, including two putative hydrophobic regions in TGB2 at amino acid positions 12–30 and 72–101 and one N-terminal hydrophobic region in TGB3 at amino acid positions 1–24, consistent with their putative roles as membrane-associated movement proteins. In addition, the ORF5-encoded coat protein contained an arginine-rich basic region, RPRLRRT, at amino acid positions 76–82, whereas the ORF6-encoded 11K protein contained a cysteine-rich motif, C-X2-C-X12-C-X4-C, at amino acid positions 59–80, corresponding to nucleotide positions 8304–8369. These conserved motifs and predicted functional regions further support the genomic organization of AnCV as typical of members of the genus *Carlavirus*.

### 3.3. Sequence Comparison of AnCV with Other Carlavirus Members

To determine the relationship between AnCV and other members of the genus *Carlavirus*, the complete genome nucleotide sequence, as well as the nucleotide and amino acid sequences of the polymerase gene and CP, were compared with those of representative *Carlavirus* members available in the GenBank database (Table 2).

Based on nucleotide and amino acid sequence identity comparisons between AnCV and other members of the *Carlavirus* (Table 2), the overall nucleotide identity of the genome ranged from 44.5 to 57.8%, with the highest identity (57.8%) observed with *potato virus H* (GenBank accession number: JQ904630.1). At the polymerase gene level, the highest nucleotide and amino acid identities were 59.4% (GenBank accession number: AM493895.2) and 46.5% (GenBank accession number: EU162589.1), respectively. In addition, the CP, an important molecular marker for species classification within the genus *Carlavirus*, showed maximum nucleotide and amino acid identities of 60.0% and 55.8%, respectively, compared with those of phlox virus B (GenBank accession number: EU162589.1).

According to the species demarcation criteria established by the ICTV for the genus *Carlavirus*, viruses are considered distinct species if the nucleotide sequence identity of the CP or polymerase gene is <72% or if the corresponding amino acid identity is <80%. In this study, the highest sequence identities between AnCV and known *Carlavirus* members (CP nt 60.0%, CP aa 55.8%; polymerase gene nt 59.4%, polymerase gene aa 46.5%) were significantly below these thresholds, providing strong evidence that AnCV is a novel species within the genus *Carlavirus*. Based on this molecular evidence, the virus identified in *A. dahurica* was tentatively designated as *Angelica carlavirus virus* (AnCV).

### 3.4. Phylogenetic Analysis of AnCV Isolates

**Figure 2:**
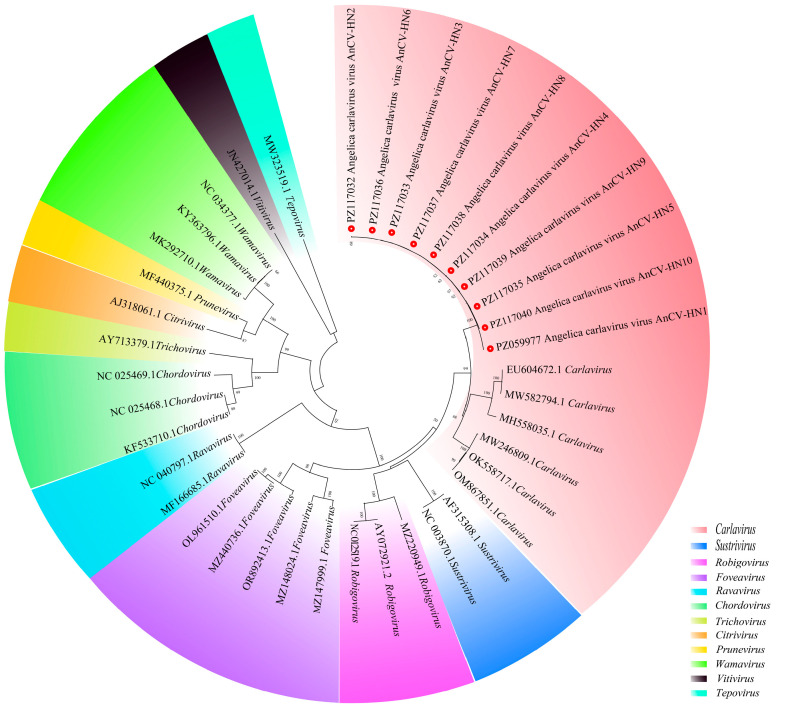
Phylogenetic tree constructed with members of Betaflexiviridae and the 10 full sequences from this study. Note: ● sequences obtained in this study.

A total of 29 related viral sequences were identified by a BLAST search of the complete genome sequence of AnCV in the NCBI database, all of which belonged to the family Betaflexiviridae. Overall, this result confirmed that the virus identified in this study is a member of Betaflexiviridae. After removing redundant and incomplete sequences, 16 full-length genome sequences of representative *Betaflexiviridae* members were selected for analysis. In addition, sequences from other genera within the family were retrieved. To determine the taxonomic position of the virus, we constructed a phylogenetic tree using sequences of representative *Betaflexiviridae* members and the complete genome sequence obtained in this study, with *Foveavirus* as the outgroup (Figure 1). As shown in the phylogenetic tree, AnCV clustered with members of the genus *Carlavirus* and formed a distinct branch (Figure 2). Overall, these results indicate that the virus shares a high degree of sequence similarity with *Carlavirus* members and exhibits a more distant evolutionary relationship with viruses from other genera within the family *Betaflexiviridae*, such as *Foveavirus*, *Trichovirus*, and *Vitivirus*.

**Figure 3:**
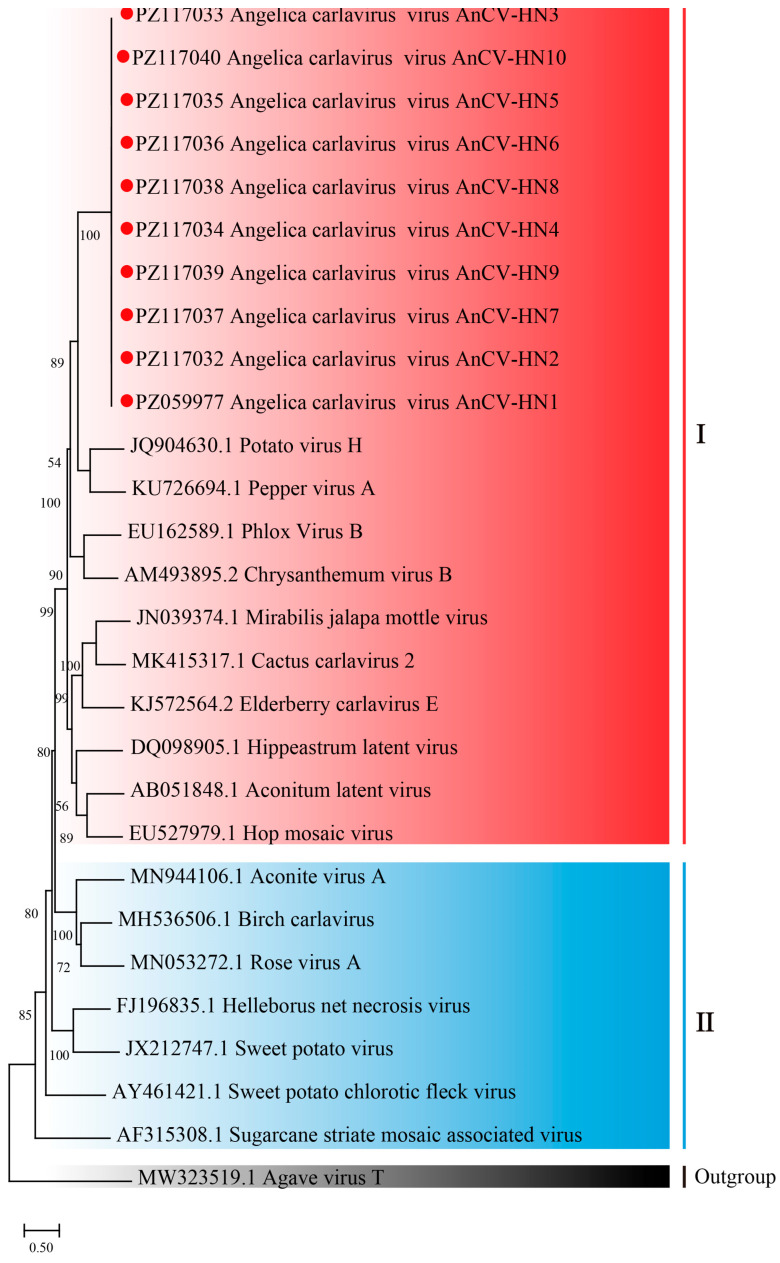
Phylogenetic tree of polymerase genes. Note: ● sequences obtained in this study; I: Subgroup I; II: Subgroup II.

**Figure 4:**
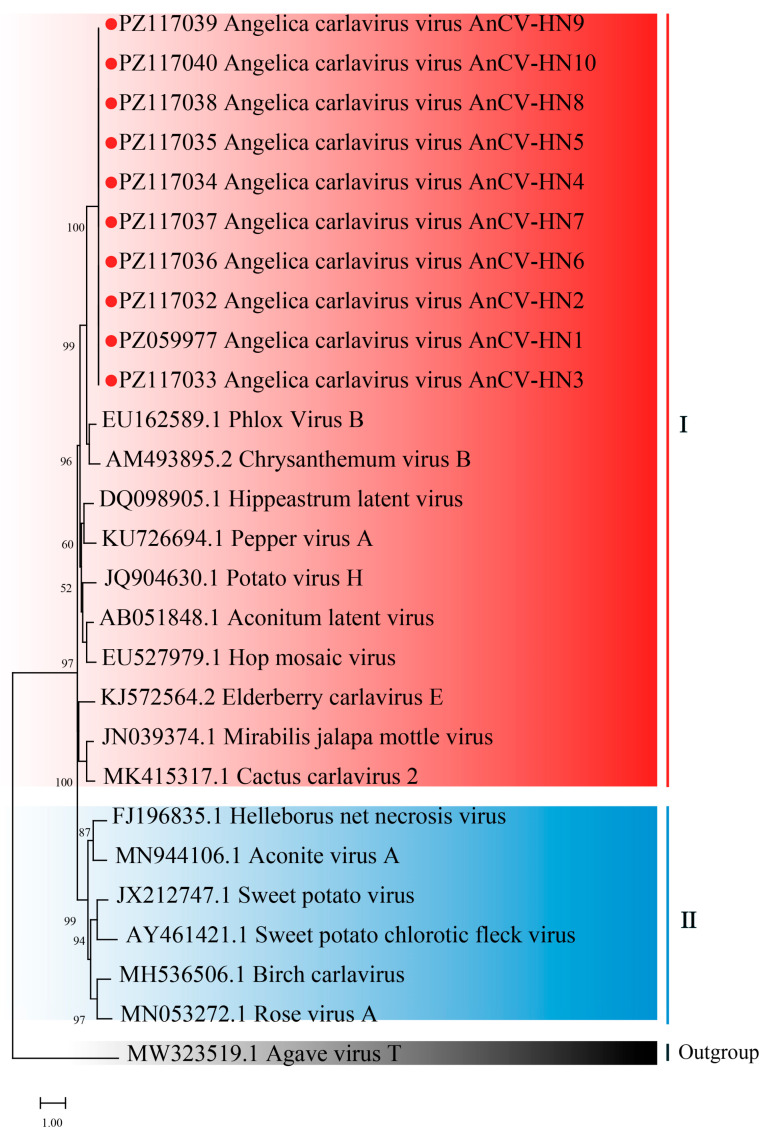
Phylogenetic tree of the coat protein. Note: ● sequences obtained in this study; I: Subgroup I; II: Subgroup II.

To further determine the taxonomic status of AnCV, phylogenetic analyses were performed using MEGA version 11.0 based on the amino acid sequences of the polymerase gene (Figure 3) and CP (Figure 4), in comparison with other members of the genus *Carlavirus*. Phylogenetic tree analysis revealed that the 10 AnCV isolates were closely related to *potato virus H* (GenBank accession number: JQ904630.1) and clustered within subgroup I (all members of the genus *Carlavirus* are divided into two subgroups, I and II) (Figure 2). Based on phylogenetic analyses of the complete genome, polymerase gene, and CP sequences, the AnCV isolates identified in this study were classified within subgroup I of the genus *Carlavirus*.

### 3.5. Recombinant Analysis Results of the AnCV Isolate

Recombination plays an important role in viral evolution and the emergence of new viruses. To investigate the evolutionary characteristics of the 10 AnCV isolates, recombination analysis was performed on complete genome sequences using RDP version 4.0. Notably, recombination occurred in the AnCV- HN1 isolate, with AnCV-HN9 and AnCV-HN10 as the major and minor parents, respectively (Table 3). Importantly, this recombination event was confirmed by seven methods (RDP, GENECONV, BootScan, MaxChi, Chimaera, SiScan, and 3SEQ), with *p*-values of 3.318 × 10^−6^, 2.708 × 10^−6^, 5.732 × 10^−6^, 2.596 × 10^−5^, 2.625 × 10^−8^, 2.913 × 10^−6^, and 6.902 × 10^−7^, respectively. In addition, recombination was detected in the AnCV-HN2 isolate, with AnCV-HN9 and AnCV-HN10 as major and minor parents, respectively. Although GENECONV identified a recombination signal among the seven detection methods, the *p*-value was greater than 10^−5^ (1.130 × 10^−3^), indicating that this event did not meet the criteria for a statistically significant recombination event.

### 3.6. RT-PCR Detection of AnCV in A. dahurica Samples

To assess the field occurrence of AnCV in *A. dahurica*, 280 leaf samples were screened with specific primers. AnCV was detected in 119 samples, corresponding to a detection rate of 42.58%. Ten PCR amplicons were randomly chosen for sequencing, and pairwise comparison showed 99.1–100% sequence identity among them.

## 4. Discussion

The genus *Carlavirus*, belonging to the family *Betaflexiviridae*, comprises an important group of plant viruses with a wide host range and global distribution [26]. To date, members of this genus have been reported in various economically important crops and ornamental plants, including *Solanum tuberosum* L. [27], *Humulus lupulus* [28], *Trifolium pratense* L. [29], and strawberry [30]. In recent years, an increasing number of novel members of the genus *Carlavirus* have been identified owing to the rapid development of high-throughput sequencing technologies, and their genetic diversity and evolutionary relationships have attracted growing attention. In this study, the near-complete genome sequence of a carlavirus infecting *A. dahurica* was obtained using high-throughput sequencing, RT-PCR, and RACE techniques. In addition, the taxonomic status of the virus was determined using sequence identity and phylogenetic analyses. Notably, this virus exhibited significant genetic divergence from previously reported carlaviruses, indicating that the isolate is a novel species within the genus *Carlavirus* and was tentatively designated *Angelica carlavirus virus* (AnCV).

AnCV has a genome structure consistent with that of several newly reported members of the genus *Carlavirus*. For example, a novel carlavirus identified in chrysanthemum in China contains six ORFs and is phylogenetically classified within the genus *Carlavirus* [31]. Similarly, soybean carlavirus 1 (SCV1), discovered in soybean in the United States, exhibits the typical six-ORF genome organization [32]. In contrast, grapevine carlavirus 1, identified in grapevines in Greece, contains only five ORFs, lacking ORF6, which is present in some *carlavirus* [22]. Collectively, these results indicate that although the overall genome structure within the genus *Carlavirus* is relatively conserved, there are still some variations in genomic organization among different members, particularly regarding the presence or absence of ORF6, which may reflect structural diversification across different hosts and evolutionary lineages of the virus. In addition, the retention of a complete six-ORF organization in AnCV suggests that its genome structure is more similar to that of the typical members of the genus than to those with simplified genomic architectures.

Importantly, the discovery of AnCV further expands the known host range of viruses within the genus *Carlavirus* and suggests that medicinal plants in the Apiaceae family are important natural reservoirs that have long been overlooked. Previous studies have shown that *carlavirus* can infect several economically important crops, ornamental plants, and perennial woody plants, including hydrangea, hellebore, grapevine, hop, soybean, and chrysanthemum, demonstrating considerable host diversity and broad geographic distribution [19,20,22]. However, this study revealed that the carlavirus AnCV can infect the medicinal plant *A. dahurica* (Apiaceae), thereby enriching the known distribution of *carlavirus* in medicinal plants and providing novel insights into host adaptation and ecological dissemination within this genus. In addition, the cross-family host distribution pattern provides important evidence for understanding the evolution of carlaviruses. Phylogenetic studies have shown that viruses in this genus do not cluster strictly according to the taxonomic relationships of their host plants but instead exhibit complex intermingled distributions across different plant families, suggesting that host switching or host expansion may be one of the key mechanisms driving their evolution [33]. Differences among host plants in cellular environments, antiviral defense mechanisms, and cultivation ecological niches [34] may impose distinct selective pressures on key viral functional proteins, such as replicase, movement protein, and CP, thereby promoting viral sequence divergence, population variation, and the emergence of new species [35,36]. Similarly, recent studies on the global population structure of potato virus S revealed the joint influence of host factors, geographic dispersal, and population genetic processes on the evolution of *carlavirus* [33]. Collectively, these findings indicate that the emergence of AnCV may be closely associated with long-term host adaptation, regional transmission, and sustained selection in specific ecological environments.

Although AnCV is classified within the *Carlavirus* subclade I, it forms an independent branch, which is consistent with reports of several newly identified members from different hosts. A newly identified carlavirus from chrysanthemum in China [37] and soybean carlavirus 1 (SCV1) from soybean in the United States of America [32] both exhibit the characteristic pattern of clear classification within the genus, forming distinct branches at the species level. Similarly, grapevine carlavirus 1 formed an independent branch in phylogenetic trees based on replicase and CP sequences. Overall, these results suggest that the emergence of new species within the genus *Carlavirus* is typically not due to minor variations from known viruses, but rather to the accumulation of sufficient sequence divergence within a conserved genomic framework, leading to the formation of stable and reproducible independent lineages [22]. Although AnCV shows relatively close phylogenetic relationships with members such as *potato virus H*, analyses at the whole-genome, polymerase gene, and CP levels consistently indicate that it is not an isolate of any known species but instead represents a novel member with an independent evolutionary status.

In this study, recombination signals were detected in some AnCV isolates, suggesting that recombination may contribute to the generation of genetic variation within the population. Recombination is considered an important mechanism driving lineage diversification and adaptive evolution in members of the genus *Carlavirus* and family Betaflexiviridae. Previous studies have analyzed the global dispersal and population genetic structure of *carlavirus*, such as *potato virus S*, and have also reported evidence of recombination and genetic rearrangements in other members [38]. Although the recombination events detected in this study require further validation using additional isolates and broader geographic sampling, the current results indicate that AnCV is not a genetically homogeneous virus population and that a certain degree of differentiation may have already occurred within its populations.

Furthermore, the detection rate of AnCV in 280 *A. dahurica* samples was 42.58%, indicating a relatively high incidence in the surveyed region of this study. Viruses of the genus *Carlavirus* are typically transmitted by aphids in a non-persistent manner, and some members can also spread through mechanical contact or propagation materials [39]. Therefore, the field prevalence of AnCV in medicinal plants, such as *A. dahurica*, is likely associated with a combination of factors, including vector insect activity, infected propagation materials, and cultivation practices.

Compared with ornamental plants, food crops, and fruit trees, the viral diversity in medicinal plants has long been understudied. Previous studies on chrysanthemum in China, soybean in the United States of America, grapevine in Greece, and hibiscus in Colombia indicate that host plants that have traditionally received limited attention may harbor abundant *Carlavirus* or related viral resources. Notably, the identification of AnCV in *A. dahurica* suggests that medicinal plant cultivation systems may be important settings for the ongoing discovery of novel plant viruses.

Although this study systematically conducted molecular identification and taxonomic analysis of AnCV, several limitations remain. For instance, the current samples were mainly collected from major production areas in Henan Province, which is insufficient to reflect the distribution pattern and population structure of AnCV on a broader geographical scale. Although a relatively high detection rate was observed in the field, there is still a lack of direct evidence regarding the actual impact of AnCV on the yield, accumulation of active compounds, and medicinal quality of *A. dahurica*. Future studies should expand epidemiological investigations to wider regions and further elucidate the biological characteristics, evolutionary history, and potential impact of AnCV on *A. dahurica* production.

In summary, AnCV possesses the typical genomic features of the genus *Carlavirus* but shows significant divergence from known members at key taxonomic loci, such as the polymerase genes and CP, and forms an independent branch in phylogenetic analyses. Combined with related reports from different host plants in countries, including China, the United States, Greece, New Zealand, and Italy, the discovery of AnCV expands the known host range and geographic distribution of the genus *Carlavirus* and suggests that medicinal plants may harbor several yet-to-be-characterized *Carlavirus* species. Overall, these findings provide a new theoretical basis for future studies on viral disease monitoring in *A. dahurica*, germplasm health evaluation, and disease control strategies.

## 5. Conclusions

In this study, high-throughput sequencing, RT-PCR, and 5′/3′ RACE were employed to successfully obtain the complete genome sequence of a virus infecting *A. dahurica*. Notably, the genome of the virus is 8562 nt in length and exhibits the typical genomic organization of the genus *Carlavirus*, containing six ORFs encoding a replicase, triple gene block proteins (TGB1, TGB2, and TGB3), a CP, and a nucleic acid-binding protein (NABP).

Based on molecular characteristics and taxonomic criteria, the sequence identities of this virus were significantly below the species demarcation thresholds established by the ICTV. Combined with phylogenetic analysis, the virus was identified as a novel species of the genus *Carlavirus* and tentatively named AnCV. In addition, the detection rate of AnCV in field-collected *A. dahurica* samples reached 42.58%, indicating a relatively high incidence in production settings and suggesting that it may pose a potential threat to the yield and quality of this medicinal plant species.

In summary, this study is the first to identify and systematically characterize a novel *carlavirus* that infects *A. dahurica*. This study enriches the available data on the genetic diversity of this genus, expands its known host range, and provides an important theoretical foundation and technical support for the diagnosis and detection, epidemiological research, and green control of viral diseases in *A. dahurica*.

## Figures and Tables

**Table 1:** Primers for *Angelica carlavirus Virus* (AnCV) whole genome amplification.

Virus Primer	Primer Sequences (5′–3′)	Product Size/bp
1-AnCV-F	TAGAGATTTGATTTCGTCAAGTAC	1500
1-AnCV-R	CCTACCTCAAAGATATCCTCATCGTC	
2-AnCV-F	GATCAATAGTAGAGAGTGGGCATTG	1700
2-AnCV-R	GCTATCAGGTCTATGTATCCAGG	
3-AnCV-F	GTTGTTCATTAGTTCAGAACACTC	1900
3-AnCV-R	CATAACGGCCAGCTCAAAAGCCAC	
4-AnCV-F	GTTGTATGGCCAATTCCTGCTG	1110
4-AnCV-R	CTACTAACACATCCATATACG	
5-AnCV-F	GATCAATGATTATATCTTCATC	2000
5-AnCV-R	GGTCTACCGATCTCCAACCCAGCG	
6-AnCV-F	GTATACACCTGAATTCATAGAACTC	1300
6-AnCV-R	CCCTATAACACCTATGACAGCG	
5′RACE-AnCV-R	CCTTATCATAGAATTAATATCATCAGTCTTGATAACCAGGTCCGC	
3′RACE-AnCV-F	CCTCTGGACATTTGCTTTGTGATTTTAAATAAAGGCTAT	

**Table 2:** Homology comparison results with other *Carlavirus* viruses.

GenBank	Virus Name	Genome Sequence (nt%)	Polymerase Genes (nt/aa%)	CP (nt/aa%)
KU726694.1	Pepper virus A	54.6	54.7/39.8	55.8/48.7
MN944106.1	Aconite virus A	48.0	48.9/38.3	45.8/33.0
MN053272.1	Rose virus A	49.3	50.0/39.4	43.3/31.5
MK415317.1	Cactus carlavirus 2	48.6	49.9/36.4	52.0/37.2
EU162589.1	Phlox virus B	54.1	56.9/46.5	60.0/55.8
MH536506.1	Birch carlavirus	49.9	50.3/39.7	45.7/31.5
JQ904630.1	Potato virus H	57.8	55.2/41.3	55.0/47.6
AY461421.1	Sweet potato chlorotic fleck virus	44.5	46.3/36.4	44.2/29.6
AM493895.2	Chrysanthemum virus B	52.2	59.4/44.8	57.7/54.7
KJ572564.2	Elderberry carlavirus E	51.4	53.8/44.8	49.8/40.4
EU527979.1	Hop mosaic virus	53.7	53.8/43.8	54.5/46.4
JX212747.1	Sweet potato virus	50.1	49.9/36.6	48.3/34.1
AB051848.1	Aconitum latent virus	53.9	53.8/42.5	55.7/49.8
DQ098905.1	Hippeastrum latent virus	53.9	54.6/42.1	54.1/44.2
JN039374.1	Mirabilis jalapa mottle virus	50.5	52.5/43.9	52.4/41.9
FJ196835.1	Helleborus net necrosis virus	54.7	52.1/41.6	49.8/33.3

**Table 3:** RDP4.0 recombination detection results.

Serial Number	Recombinant Isolate	Primary Parent	Secondary Parent	Testing Method						
				R	G	B	M	C	S	T
1	AnCV-HN1	AnCV-HN9	AnCV-HN10	3.318 × 10^−6^	2.708 × 10^−6^	5.732 × 10^−6^	2.596 × 10^−5^	2.625 × 10^−8^	2.913 × 10^−6^	6.902 × 10^−7^
2	AnCV-HN2	AnCV-HN9	AnCV-HN3	2.251 × 10^−7^	1.130 × 10^−3^	1.253 × 10^−6^	3.198 × 10^−10^	1.871 × 10^−10^	6.746 × 10^−11^	2.835 × 10^−10^

## Data Availability

The raw data has been uploaded to SRA NCBI (PRJNA1465610).

## Abbreviations

The following abbreviations are used in this manuscript:

AnCV	*Angelica carlavirus virus*
CP	Coat protein
ICTV	International Committee on Taxonomy of Viruses
